# Clinical, Neurophysiological, Radiological, Pathological, and Genetic Features of Dysferlinopathy in Saudi Arabia

**DOI:** 10.3389/fnins.2022.815556

**Published:** 2022-02-22

**Authors:** Norah Alharbi, Rawan Matar, Edward Cupler, Hindi Al-Hindi, Hatem Murad, Iftteah Alhomud, Dorota Monies, Ali Alshehri, Mossaed Alyahya, Brian Meyer, Saeed Bohlega

**Affiliations:** ^1^Department of Clinical Science, College of Medicine, Princess Nourah Bint Abdulrahman University, Riyadh, Saudi Arabia; ^2^Salmaniya Medical Complex, Manama, Bahrain; ^3^Department of Neuroscience, King Faisal Specialist Hospital, and Research Center, Jeddah, Saudi Arabia; ^4^Department of Pathology and Laboratory Medicine, King Faisal Specialist Hospital and Research Center, Riyadh, Saudi Arabia; ^5^Department of Neuroscience, King Faisal Specialist Hospital and Research Center, Riyadh, Saudi Arabia; ^6^Department of Genetics, King Faisal Specialist Hospital and Research Center, Riyadh, Saudi Arabia

**Keywords:** dysferlinopathy, limb-girdle muscular dystrophies (LGMD), LGMD2B, Miyoshi myopathy, dysferlin, DYSF gene, Saudi Arabia, neurophysiological profile

## Abstract

**Background:**

To characterize the phenotypic, neurophysiological, radiological, pathological, and genetic profile of 33 Saudi Arabian families with dysferlinopathy.

**Methods:**

A descriptive observational study was done on a cohort of 112 Saudi Arabian families with LGMD. Screening for the Dysferlin (DYSF) gene was done in a tertiary care referral hospital in Saudi Arabia. Clinical, Neurophysiological, Radiological, Pathological, and Genetic findings in subjects with dysferlin mutation were the primary outcome variables. Statistical analysis was done by Epi-info.

**Results:**

33 out of 112 families (29.46%) registered in the LGMD cohort had Dysferlinopathy. 53 subjects (28 males, 52.83%) from 33 families were followed up for various periods ranging from 1 to 28 years. The mean age of onset was 17.79 ± 3.48 years (Range 10 to 25 years). Miyoshi Myopathy phenotype was observed in 50.94% (27 out of 53), LGMDR2 phenotype in 30.19% (16 out of 53), and proximodistal phenotype in 15.09% (8 out of 53) of the subjects. Loss of ambulation was observed in 39.62% (21 out of 53 subjects). Electrophysiological, Radiological, and histopathological changes were compatible with the diagnosis. Mean serum Creatinine Kinase was 6,464.45 ± 4,149.24 with a range from 302 to 21,483 IU/L. In addition, 13 dysferlin mutations were identified two of them were compound heterozygous. One founder mutation was observed c.164_165insA in 19 unrelated families.

**Conclusion:**

The prevalence of Dysferlinopathy was 29.46% in the native Saudi LGMD cohort. It is the most prevalent subtype seconded by calpainopathy. The clinical course varied among the study subjects and was consistent with those reported from different ethnic groups. One founder mutation was identified. Initial screening of the founder mutations in new families is highly recommended.

## Introduction

“Limb-girdle muscular dystrophies” (LGMD) are a diverse group of rare progressive genetic disorders, characterized by progressive weakness of muscles of pelvis and muscles of the shoulder girdle (Vissing, 2016; Liewluck and Milone, 2018). Globally, it is the fourth most common muscular dystrophy (Mundinger, 1965; Mah et al., 2014). There are various types of LGMD, classified based on the mutations in the responsible genes. Dysferlinopathy are muscular dystrophies with an autosomal recessive inheritance. They are characterized by diverse mutations in the dysferlin (DYSF) gene found on chromosome 2p13 (Bashir et al., 1994). DYSF gene is involved in limb-girdle muscular dystrophy type 2B (LGMD2B), \LGMDR2 (Straub et al., 2018), Miyoshi myopathy disease type 1 (MMD1), and a rare form of distal anterior compartment myopathy (DACM) (Miyoshi et al., 1986; Liu et al., 1998; Illa et al., 2001; Urtizberea et al., 2008). MMD1 is characterized predominantly by the involvement of the calf muscles and high elevation of serum creatine kinase (CK) levels (Miyoshi et al., 1986). LGMDR2/LGMD2B predominantly affects the proximal muscles, while DACM mainly causes distal myopathy with anterior tibial onset (Urtizberea et al., 2008). The DYSF gene encodes a 230 kilodalton protein, dysferlin. This protein is usually expressed at the level of the sarcolemma in skeletal muscle. The affected protein is absent in patients affected with Dysferlinopathy (Weiler et al., 1999; Bansal et al., 2003; CDSI, 2010). The mechanism by which it causes muscle fibers necrosis is still not entirely understood. Speculative theories include impaired membrane repair (Han, 2011; Matsuda et al., 2015), inflammation (McNally et al., 2000; Han, 2011), and fat deposition (Grounds et al., 2014) in dysferlin deficient muscles. The severity of the disease ranges from asymptomatic carriers of mutation to patients presenting with elevated serum CK levels (Dabby et al., 2006), intolerance to exercise, to severe functional disability (Petersen et al., 2015). Interestingly, identical mutations in the same family could result in phenotypic variation with a different group of affected muscles (Weiler et al., 1999; Nakagawa et al., 2001). The pathogenesis for the presentation of two phenotypes resulting from a single gene mutation still remains poorly understood. Dysferlinopathy can account for up to 30% of progressive recessive muscular dystrophies in some geographical regions (Urtizberea et al., 2008). LGMDR2 is the second most common LGMD in many areas of the world (Mahmood and Jiang, 2014). The overall prevalence of LGMD has been estimated at around 1.63 per 100,000 population (Mundinger, 1965). LGMDR2 accounts for nearly 3–19% of all LGMDs across the world (Moore et al., 2006; van der Kooi et al., 2007; Guglieri et al., 2008; Lo et al., 2008). Ethnic clusters (Dincer et al., 1997; Linssen et al., 1997; Argov et al., 2000; Nakagawa et al., 2001; Cagliani et al., 2003; Takahashi et al., 2003; Zatz et al., 2003; Vilchez et al., 2005; Moore et al., 2006; Sveen et al., 2006; Leshinsky-Silver et al., 2007; Guglieri et al., 2008; Lo et al., 2008; Fanin et al., 2009; Norwood et al., 2009; Hayashi et al., 2010; Gomez-Diaz et al., 2012; Mahmood and Jiang, 2014; Xi et al., 2014; Petersen et al., 2015) have been reported in Jews from Libya (Argov et al., 2000) and the Caucasus region (Leshinsky-Silver et al., 2007), as well as in the Italian (Cagliani et al., 2003; Guglieri et al., 2008; Fanin et al., 2009), Spanish (Vilchez et al., 2005), Swiss (Petersen et al., 2015), Mexican (Gomez-Diaz et al., 2012), and Japanese populations (Takahashi et al., 2003; Hayashi et al., 2010). Relative proportion of LGMDR2 among LGMD varies from 1.7% in Denmark to 41% in Mexico (Mahmood and Jiang, 2014). To the best of our knowledge, no study has been published on genetically proven dysferlinopathy in Saudi Arabia. The present study is carried out to describe the clinical, neurophysiological, radiological, pathological, and genetic characteristics of 33 Saudi Arabian families with dysferlin gene mutations.

## Materials and Methods

A descriptive cross-sectional record-based observational study was done on a cohort of 112 Saudi Arabian families with LGMD. King Faisal Specialist Hospital & Research Center (KFSH&RC) is a major tertiary care center in Saudi Arabia. It receives tertiary referrals from other regional hospitals in the kingdom. KFSH&RC Neuromuscular registry was established in 2005, funded by King Abdulaziz City for Science and Technology (KACST) Grant no (08-MED498-20). The registry includes all cases of LGMD diagnosed according to European Neuromuscular Centre (ENMC) (Bushby, 1995; Bushby and Beckmann, 1995). The study was also approved by the hospital Research Advisory Council (RAC) (Project no. 2070 005). Cases of Dysferlinopathy were selected from the registry. They were identified in 29 families by the presence of dysferlin mutations, confirmed between February 2005 and February 2021 and in the four remaining families according the pathology and immunohistochemistry testing. All the subjects provided their written informed consent for genetic analysis. Medical records of the patients were reviewed. If family history was positive, other affected members of the family were recruited. The clinical data collected included gender, age, province of residence, culture (Bedouin or Nomad vs. Urban), family pedigree, age at onset of symptoms (mainly the onset of the muscle weakness), main phenotype, follow-up period, and functional status.

### Chart Review and Laboratory Investigations

Detailed review of blood investigations, including serum biochemical parameters and hepatic profile, was done. Cardiac investigations, mainly electrocardiography and echocardiography, were performed. Neurophysiological tests (electromyography), muscle MRI, and muscle biopsy data were also reviewed if available.

### Imaging

Muscle MRI was performed at the time of the diagnosis with a 1.5 Tesla Magnetom, Siemens, Erlangen MRI scanner. Thigh and leg MRI sections were obtained from axial planes utilizing T1-weighted spin-echo, T2-weighted spin-echo, and a short-time inversion recovery (STIR) sequence. The involvement of all thigh and lower leg muscles were evaluated according to increased signal intensity, which is mainly due to increased fatty infiltration. The pattern of muscle involvement was categorized according to anatomic distribution. The thigh muscles were divided into three compartments: anterior, medial, and posterior compartments. The leg muscles were divided into two compartments: anterolateral and posterior compartments. STIR sequence, a fat suppression technique, was used to demonstrate myoedema.

### Pathology

Open muscle biopsies were performed under local anesthesia from the deltoid, quadriceps, or gastrocnemius muscles. Then the collected specimens were frozen in isopentane and cooled at –160°C with liquid nitrogen. Then 4-millimeter serial cryostat sections were processed for enzyme histochemistry. Hematoxylin and Eosin (H&E), Periodic Acid-Schiff (PAS), Modified Gomori trichrome, and oil red O were the routinely used stains as well as a battery of enzyme histochemical stains including reduced nicotinamide adenine dinucleotide-tetrazolium reductase (NADH-TR), succinic dehydrogenase (SDH), cytochrome oxidase (COX), acid phosphatase, alkaline phosphatase, non-specific esterase and adenosine triphosphatase at pH 4.3, 4.6 and 9.4. In addition, immunohistochemical detection of dystrophin, sarcoglycans and dysferlin was sought among other available cytoskeletal proteins. A total of 37 proper muscle biopsies were available for review by two certified neuropathologist.

### Genetic Analysis

All genes reported to cause LGMD was studied utilizing genome-wide linkage, homozygosity mapping, and whole-exome sequencing. A summary of the methodology followed is described below.

Initially, extraction of genomic DNA was done from a peripheral blood sample by standard procedures (Flexi Gene DNA Handbook, Qiagen). Then these samples were quantified spectrophotometrically. They are stored at −20°C. Genotyping, and homozygosity mapping were performed in all the individuals, including affected and unaffected, from multiplex and simplex families. They were then genotyped using the Affymetrix Axiom array (Affymetrix, Santa Clara, CA, United States) using the manufacturer’s protocol^1^. Then using autoSNPa^2^, the resulting genotypes were analyzed for shared runs of homozygosity (ROH). Multiplex families underwent Whole Exome Sequencing (WES). Ion Proton AmpliSeq libraries were built from one hundred nanograms of each DNA sample. Then, in twelve separate wells, DNA was amplified using Exome Primer Pools, AmpliSeq HiFi mix (Thermo Fisher, Carlsbad, CA, United States), and 10 amplification cycles. After that, the twelve PCR pools were combined in one well. They were subjected to primer digestion by incubating with FuPa reagent (Thermo Fisher, Carlsbad, CA, United States). Then the amplified Exome targets were ligated with Ion P1 and Ion Xpress Barcode adapters. Libraries were quantified using qPCR, after purification with the Ion Library Quantification Kit (Thermo Fisher, Carlsbad, CA, United States). The prepared exome library was further used for emulsion PCR on an Ion OneTouch System. Templated Ion Sphere particles were enriched using Ion OneTouch ES. The template-positive Ion PI Ion Sphere particles were processed for sequencing on the Ion Proton instrument. For every sequencing run, around 15--17 Gb of the sequence was generated. Finally, the reads were mapped to UCSC hg19^3^. The variants were identified using the Saudi Human Genome Program (SHGP) pipeline (Saudi Mendeliome, 2015).

### Statistical Methods

Descriptive statistics were used to analyze data in accordance with the study’s objectives. Data were expressed as the mean, 95% confidence interval (CI; lower and upper bounds), median, minimum and maximum, and percentage, where appropriate. Data was also represented using appropriate diagrams like bar diagrams, pie diagrams. Data will be analyzed by using EPI info.

## Results

The summary of baseline characteristics of the study population is described in Table 1. Socio-demographic, Clinical, Neurophysiological, Radiological, Pathological, and Biochemical characteristics of subjects with Dysferlinopathies in Saudi Arabia are described in Supplementary Table 1.

**TABLE 1:** Summary of baseline parameter in the study population (*N* = 53).

Parameter	Summary
Age	39.55 ± 8.82 (ranged 22–61)
**Gender**	
Male	28 (52.83%)
Female	25 (47.17%)
**Province**	
C (Central)	20 (37.74%)
E (East)	8 (15.09%)
S (South)	2 (3.77%)
SW (South West)	8 (15.09%)
W (West)	15 (28.30%)
**Origin**	
Bedouin	32 (60.38%)
**Family history**	
Yes	36 (67.92%)
Yes, but not genetically tested	1 (1.89%)
Age Of Onset	17.79 ± 3.48 (ranged 10–25)
Follow Up (in years)	8.57 ± 5.89 (ranged 1–28)
**Phenotype**	
LGMD	16 (30.19%)
MM	27 (50.94%)
No Symptom (hyperCKemia)	2 (3.78%)
Proximodistal	8 (15.09%)
**Functional status**	
Wheel chaired	21 (39.62%)
Ambulating	19 (35.85%)
Ambulating with aid	13 (24.53%)
Ck Level (Normal < 195 U/L)	6,464.45 ± 4,149.24 (ranged 302–21,483)
AST (Normal < 45 U/L)	91.75 ± 56.31 (ranged 27–318)

### Socio-Demographic Characteristics

**FIGURE 1:**
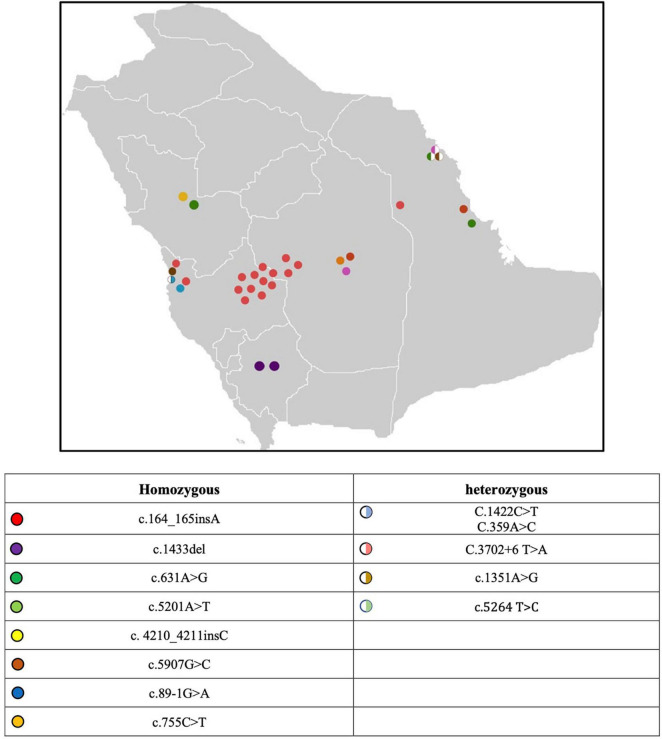
Map of Saudi Arabia with the distribution of genetic mutation.

A cohort of 112 Saudi Arabian families with LGMD were identified in the registry. 33 families (29.46%) were determined to have dysferlinopathy with 29\33 having dysferlin mutations identified. A total of 53 subjects were studied 28 (52.83%) of those subjects were males. Positive family history was observed in 67.92% (36/53). 17 subjects were sporadic and had no family history, notably most of these patients belong to highly consanguineous families. Subjects were geographically distributed in the five provinces of Saudi Arabia as shown in Figure 1: 37.74% (20/53) of subjects were identified in the Central province, 28.3% (15/53) in the western province, and 15.09% (8/53) in the south-western province and another 15.09% (8/53) in the eastern region. The majority (60.38%, 32/53) of subjects with dysferlinopathy were Bedouins (nomads).

### Clinical Characteristics

The mean age at onset of disease was 17.79 ± 3.48 years (Range 10 to 25 years). Miyoshi Myopathy phenotype was observed in 50.94% (27 out of 53), LGMDR2 phenotype in 30.19% (16 out of 53), and proximodistal phenotype in 15.09% (8 out of 53) of the subjects.

**FIGURE 2:**
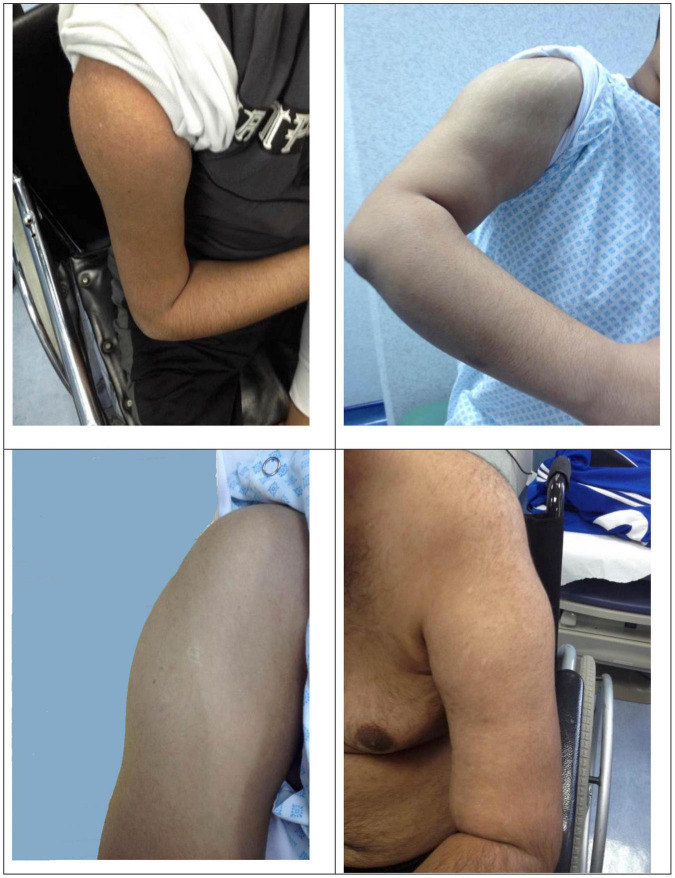
Deltoid sparing.

Two subjects (F11A, F21A) presented with incidental hyper-CK-emia with no symptoms. The initial symptoms reflected the observed phenotype in each individual. Patients with MM showed mainly difficulty in running and exercising, knee buckling, joint and back pain. On the other hand, the initial complaint in patients with LGMDR2 phenotype was related to the proximal weakness, such as difficulty standing up from a chair. There was variability in the clinical phenotype, as seen in Supplementary Table 1. Intrafamilial variability was noted in 15 families. Deltoid sparing was observed in 18 patients Figure 2. One patient had remarkably hyper-extendable knees (F23C). The average duration of follow-up was 8.57 ± 5.89 years, ranging from 1 to 28 years.

**FIGURE 3:**
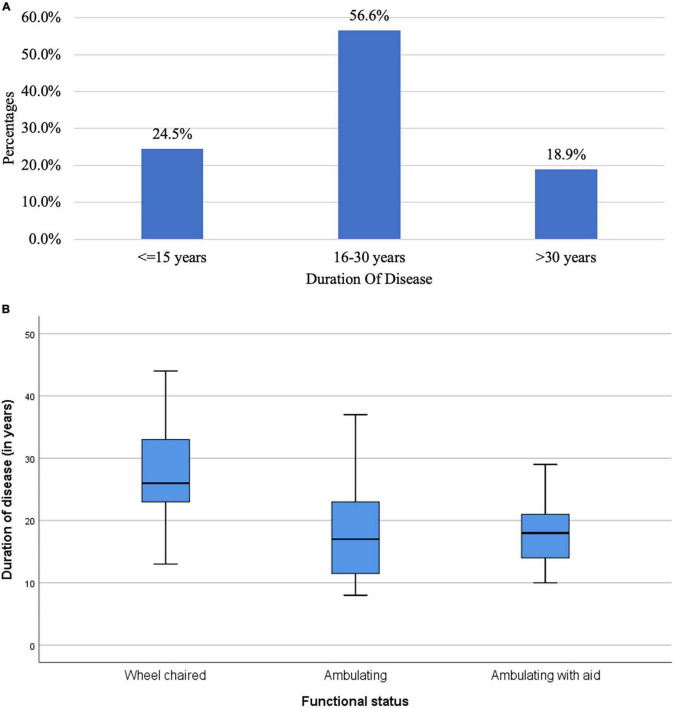
**(A)** Bar chart of duration of disease in the study population (*N* = 53). **(B)** Box plot for distribution of duration of disease across function status (*N* = 53).

With regards to functional status, 39.6% were confined to a wheelchair, 35.9% were ambulatory and 24.5% were ambulatory with aids. Figure 3A characterizes the patients based on the duration of disease. Majority (56.6%) had duration of disease between 16 to 30 years. 24.5% had disease duration not more than 15 years. Figure 3B shows the boxplot for distribution of disease across the functional status. The median duration of disease in wheel chaired patients was 26 years (Range: 13 to 44 years).

Four patients were wrongly diagnosed initially. One patient (F04A) was wrongly diagnosed with Becker dystrophy. Three patients were wrongly diagnosed for many years as inflammatory myositis and had received immunosuppressive therapy for years. (F09A, F23C, F21A). One patient (F21A) exhibited an accelerated decline in his mobility with loss of ambulation within 1 month after being wrongly diagnosed as inflammatory myopathy and managed with a high corticosteroid dose. Furthermore, all of the three patients showed worsening of the ambulatory status with no improvement after the steroid was discontinued.

### Neurophysiological, Radiological, and Biochemical Characteristics

Supplementary Table 1 shows the results of blood investigations serum creatinine kinase (CK), liver function test (LFT), EMG, radiology, and muscle biopsy findings in all subjects with Dysferlinopathy. Serum CK levels during the disease were elevated 1.5 times to 110 times the upper limit of the normal range (<195 IU/L) in all of the patients. It ranged widely between 302 to 21,483 U/L, as shown in Table 1. Liver enzyme (AST, ALT) were elevated 3–5 times the normal range in majority of patients with normal other parameter. We find a weak negative correlation between the CK, AST and ALT levels and the duration of disease (P value: 0.005) (Table 2).

**TABLE 2:** Spearman rank correlation between duration of disease (in years) and CK levels (in U/L), AST and ALT (*N* = 53).

Parameter	Spearman rank correlation (*r*_*s*_)	*P* value
CK levels (in U/L)	−0.494	<0.001
AST	−0.462	0.005
ALT	−0.358	0.027

**FIGURE 4:**
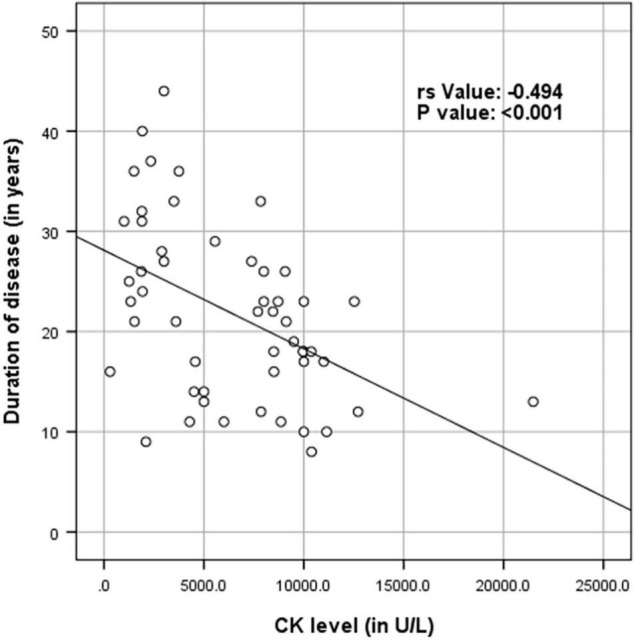
Scatter plot diagram of correlation between duration of disease (in years) and CK levels (in U/L) (*N* = 53).

**FIGURE 5:**
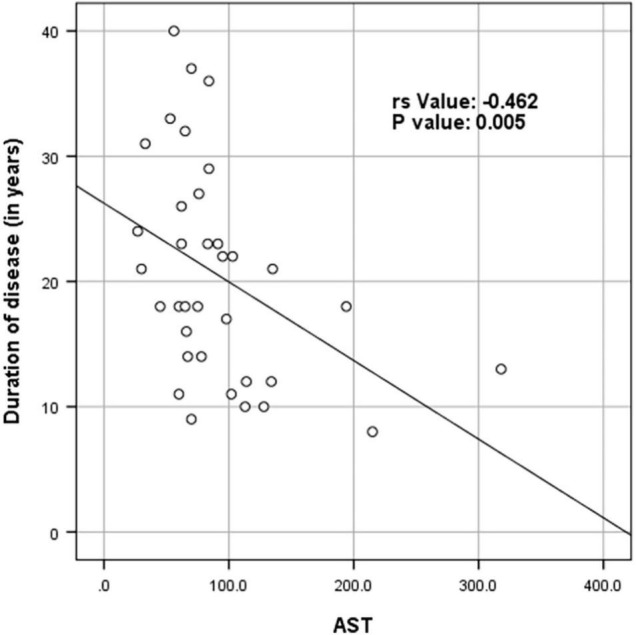
Scatter plot diagram of correlation between duration of disease (in years) and AST (*N* = 53).

**FIGURE 6:**
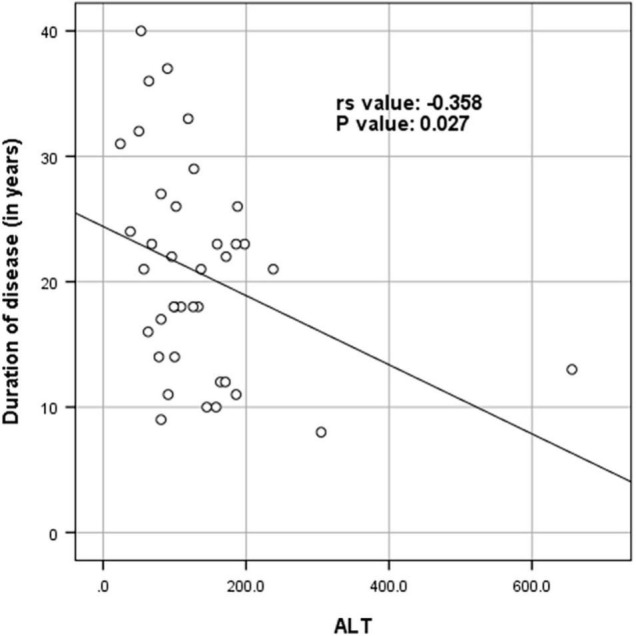
Scatter plot diagram of correlation between duration of disease (in years) and ALT (*N* = 53).

*There was a week negative correlation between ck levels (in U/L) and duration of disease (in years) (r_s_ value: −0.494, P value: < 0.001). There was a week negative correlation between AST and duration of disease (in years) (r_s_ value: −0.462, P value: 0.005). There was a week negative correlation between ALT and duration of disease (in years) (r_s_ value: −0.358, P value: 0.027) (Table 3 and Figures 4–6).*

Figures 4–6 show the correlation between disease duration in years and CK, AST, ALT levels respectively. There was a weak negative correlation between CK, AST and ALT levels (in U/L) and duration of disease (in years) as shown in Table 2.

The cardiac assessment was performed in 14 (31.8%) patients, and the results were normal except in three patients. The first patient (F9 B) had a right bundle branch block (RBBB), and his echocardiogram showed mild dilated cardiomyopathy with impaired right ventricular function. The second patient (F10 A) showed abnormal ischemic changes in the ECG. An echocardiogram for the same patient showed ischemic cardiomyopathy with an EF of 20–30%. In the third patient (F12A), the electrocardiogram showed a prolonged QT interval. All of the three patients are following up with the cardiologist for at least 4 years with stable cardiac condition. The second patient (F10A) was treated with high dose diuretic and multiple antihypertensive medications, whereas the other two patients were stable without treatment. Electromyogram (EMG) was performed in 28 (52.8%) out of 53 subjects. It showed myopathic changes in 27 subjects. Inpatient F7A, the EMG showed a mixed pattern (neurogenic and dystrophic) with companied denervation activity and definite myopathic changes (probably related to the inflammatory change).

**FIGURE 7:**
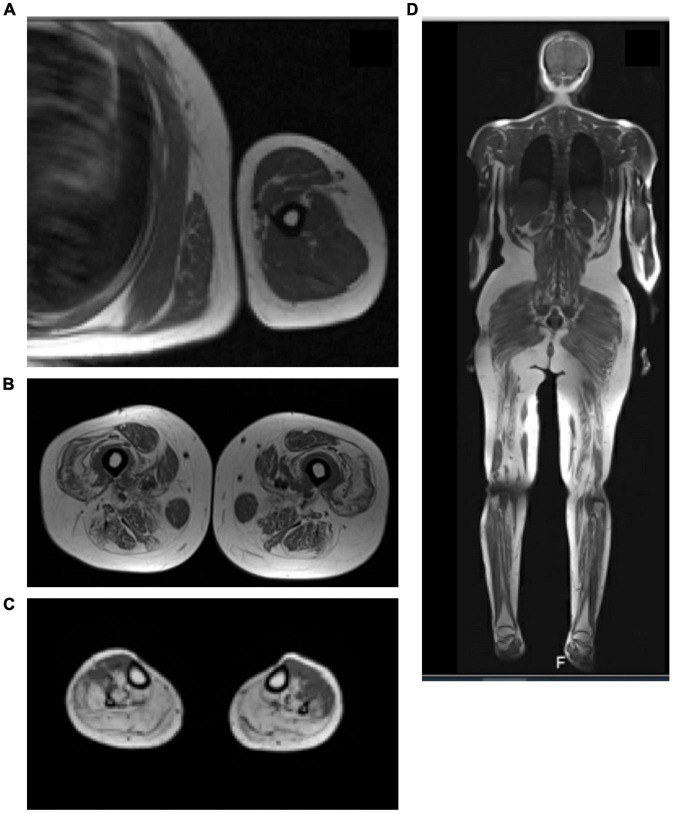
MRI of the leg with active inflammation. **(A)** MRI T1 of the upper limb showed the evidence of mild feathery muscle edematous changes involving the deltoid muscle and the biceps brachii muscle. **(B)** T1 MRI of the high muscle showed diffused fatty infiltration of the high muscle bilateral with sparing of the Sartorius muscle and relative sparing of the abductor. **(C)** T1 MRI of the legs showed significant fatty infiltration of the posterior and lateral compartments of the legs with relative sparing of the anterior compartment. **(D)** Whole body MRI scan (T1-weighted scan) showed diffused feathery edematous changes involve the thigh and leg muscle.

Magnetic Resonance Imaging (MRI) of the lower limbs was done for 21 (38.2%) subjects within varying periods from the diagnosis. It showed a variable degree of muscle atrophy, and fatty infiltrates. Edema and signs of inflammatory myositis were seen in five cases (F01A, F03E, F07A, F09A, F17A, F21A) as shown in Figure 7.

### Pathological Characteristics

**FIGURE 8:**
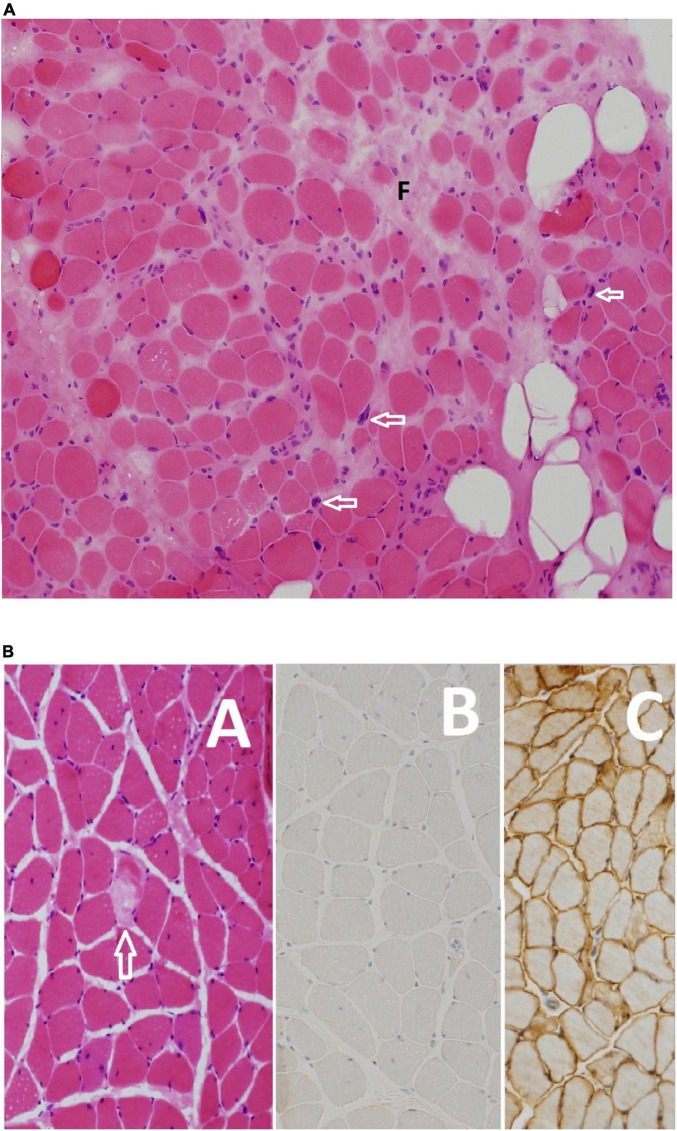
**(A)** Moderate dystrophic changes manifesting with marked variation in myofiber size and fibrosis (F), and fatty infiltration (empty rounded spaces). Note also the pyknotic nuclear clumps (arrows). Myofiber necrosis and regeneration are not shown (Hematoxylin and eosin, original magnification ×200) F09 B. **(B)** This patient’s disease manifests with scattered necrotic fibers (arrow) among non-specific myopathic changes **(A)** including variation in myofiber size nuclear internalization and split fibers. Absence of expression of dysferlin confirmed the diagnosis at the light microscopy level **(B)**. Normal expression of healthy muscle is shown in panel **(C)** for comparison [**(A)** Hematoxylin and eosin, original magnification ×200; **(B,C)** anti-dysferlin immunostain, original magnification ×200] F27 A.

Thirty-two (60.3%) patients had at least one proper and informative muscle biopsy out of the 53 patients in the study. Indications for repeating the biopsies included non-informative formalin-fixed tissue performed in peripheral hospitals (eight patients) and inconclusive findings discordant with the clinical diagnoses in six cases. Histopathological findings were reviewed for 37 out of 38 appropriate biopsies: In addition to the non-specific myopathic changes that was observed in all biopsies, 21 (57%) showed frank dystrophic changes, 13 (35%) showed variable myonecrosis (“necrotizing myopathy”) and 3 (8%) were end stage muscles. Immunohistochemical expression of dysferlin was absent in 27/32 (84%) biopsies and reduced in 5/32 (15.5%). The stain was not performed or available in 6/38 biopsies. Expression of the sarcoglycans and dystrophin proteins was reduced along with dysferlin in one biopsy (F27A); however, upon repeat the new biopsy showed absence of dysferlin and normal expression of the other proteins. Inflammation which is a typically mild, was seen in nine biopsies. Mitochondrial proliferation was also seen in nine biopsies and was marked in one (F9A). A rare rimmed vacuole was noted in one biopsy (F19A). Fiber type alterations were seen as follows: type 2 predominance, seven biopsies; type 1 predominance, three biopsies; and type 2 atrophy in four biopsies (Figure 8).

### Genetic Testing

Dysferlin mutation analysis (Exon, mutation, and consequence of mutation) of subjects with Dysferlinopathy in Saudi Arabia are summarized in Table 3.

**TABLE 3:** Mutations identified in Saudi Arabian LGMDR2 cohort: (*n* = 29).

Family number	Exon	Mutation	Consequence of mutation	Heterozygosity Type	Pathogenicity
19 families (F01, F02, F03, F07, F09, F11, F12, F13, F14, F17, F19, F20, F22, F23, F28, F 31, F30, F32, F33)	3	NM_003494.4:c.164_165insA	Frameshift	Homozygous	Pathogenic
F04	6	NM_003494.4:c.631A > G	Missense	Homozygous	Pathogenic
2 families (F05, F08)	16	NM_001130976:c.1433del	Frameshift	Homozygous	Pathogenic
F06	46	NM_001130976:c.5201A > T	Missense	Homozygous	Pathogenic
F10	39	NM_003494.4c. 4210_4211insC	Frameshift	Homozygous	Pathogenic
F15	52	NM_003494.4c.5907G > C	Missense	Homozygous	Pathogenic
F16	16	NM_003494.4:c.1422C > T	Silent	Heterozygous	Likely pathogenic
	5	NM_001130987.2:c.359A > C	Missense	Heterozygous	Likely pathogenic
F18	2	NM_001130976:c.89-1G > A	Splice site	Homozygous	Pathogenic
F21	7	NM_001130987.2:c.755C > T	Missense	Homozygous	VUS
F24	14	ENSST00000410020.8:c.1351A > G	Missense	Heterozygous	VUS
	38	NM_003494.4:c.5264 T > C	Missense		
	2	ENSST00000410020.8c.3702 + 6T > A	Intronic		

*VUS, variant of uncertain significance.*

The genetic analysis was performed for 49 (92.4%) patients, revealed 13 dysferlin mutations (Table 3). Of the forty-nine patients analyzed 47 carried homozygous DYSF variants. compound heterozygous DYSF variant were seen in two patients (F16, F24), one of them had triple heterozygous mutation. The location of the identified mutations was across the entire DYSF gene with no hot spot. c.164_165insA was the most frequent mutation among all cases seen in 28 (59.5%) patients from 19 unrelated family Figure 1. It is also, the most frequent mutation among all the phenotypes. As its seen in 59.25% (16 out 27) of the MM phenotype, 50% (8 out 16) of the LGMD2B phenotype, 37.5% (3 out 8) compound phenotype in addition to one asymptomatic (hyperCKemia) case. The second most frequent variant was c.1433delC mutation seen in three patients from two unrelated family. Regarding the consequence of the mutation we identified three frameshift mutations, one splice-site mutations, seven missense mutation, one silent mutation and one intronic mutation. The pathogenicity of three missense heterozygous variants mutation namely c.5264 T > C, c.1351A > G and c.755C > T remained of uncertain significance. Three novel variants were discovered namely c.5201A > T, c.4210_4211nsC and, c.1433del.

## Discussion

Limb-girdle muscular dystrophy includes a spectrum of a heterogeneous group of muscular dystrophy disorders (Mahmood and Jiang, 2014). Currently, there are more than 25 LGMD types that are grouped according to their genetic characteristics (KM, 1995; Mahmood and Jiang, 2014). The age at onset of the disease, severity, and progression of the disease may vary significantly from subject to subject depending on the phenotype and even maybe among the same family members depending on the mutation.

The prevalence of LGMD2B\LGMDR2 among LGMD varies from 1.7 to 41% (Mahmood and Jiang, 2014). In Saudi Arabia the most common LGMD is LGMD2B\LGMDR2 (29.46%) followed by LGMD2A (19.64%) (Alharbi et al., 2021). Moreover, the diagnosis of dysferlinopathy was establish in 33 out of the 112 families (29.46%) with LGMD. LGMDR2 phenotype was observed in 30.19% (16 out of 53) of the subjects Miyoshi Myopathy phenotype was seen in 50.94%, while proximodistal phenotype was observed in 15.09% of the subjects. Genetic findings of LGMD in 13 Saudi Arabian families were described previously in the pediatric age group by Boyden et al. (2010), But not surprisingly, none of their subjects had dysferlin gene mutation as most of the cases of dysferlinopathy presented in late teens and early twenties (Aoki and Takahashi, 2004; Boyden et al., 2010; Payam and Bönnemann, 2015). Clinical findings of seven cases of MM were described in Saudi Arabians by Cupler et al. (1998), but they did not perform genetic analysis on these subjects.

With the introduction of muscle immunochemistry and genetics, a growing number of clinical variants of dysferlinopathy became evident such as proximodistal, asymptomatic hyperCKemia and pseudometabolic disorder with exercise intolerance, myalgias and cramps but without myoglobinuria (Nguyen et al., 2005, 2007; Guglieri et al., 2008; Urtizberea et al., 2008; Paradas et al., 2010). The rate of progression to a severe clinical stage varied greatly, ranging from 12 to 35 years from the onset of the disease, independently of the initial phenotype (Guglieri et al., 2008; Paradas et al., 2010). Disease progression in dysferlinopathy is generally slow over decades, but 10–20% of MM patients nevertheless become wheelchair dependent (Urtizberea et al., 2008). The results of the present study were comparable to those found in other ethnic groups with regards to demographic and clinical presentation (Dincer et al., 1997; Linssen et al., 1997; Argov et al., 2000; Nakagawa et al., 2001; Cagliani et al., 2003; Takahashi et al., 2003; Zatz et al., 2003; Vilchez et al., 2005; Moore et al., 2006; Sveen et al., 2006; Leshinsky-Silver et al., 2007; Guglieri et al., 2008; Lo et al., 2008; Fanin et al., 2009; Norwood et al., 2009; Hayashi et al., 2010; Gomez-Diaz et al., 2012; Mahmood and Jiang, 2014; Xi et al., 2014; Petersen et al., 2015).

There was marked intra-familial phenotypic variability and heterogeneity within non-related individuals with identical dysferlin mutations in the present study. This phenotypic variability has been reported in few previous studies (Miyoshi et al., 1986; Linssen et al., 1997; Liu et al., 1998; Argov et al., 2000; Cagliani et al., 2003; Petersen et al., 2015). But, the absence of infra-familial variability has also been documented in certain ethnic groups (Takahashi et al., 2003; Vilchez et al., 2005). This implies that in addition to the genetic factors, environmental factors can also play a crucial role in the clinical heterogeneity of the disease. Dysferlin is a plasma membrane protein expressed predominantly in skeletal muscle, heart, and kidney and to a lesser extent in other tissues, including the stomach, lung, uterus, liver, spleen, and brain (Anderson et al., 1999). One case of dysferlinopathy presenting with choreic movement was reported in Japan (Takahashi et al., 2006). It is suggested that chorea may be associated with altered expression of the brain isoform of dysferlin (Takahashi et al., 2006). None of the subjects in our study showed any signs of central nervous system involvement.

One notable observation in the present study was a moderate and consistent elevation in AST and ALT levels in all the patients tested except in one (F19A). The increase in AST and ALT was mostly two to fivefold of the normal upper limits. The maximum recorded AST was 318 U/L and ALT 656 U/The source of the ALT and AST in these patients is the muscle damaged, similar to CK. Furthermore, AST and ALTenzyme abnormalities were reported in a few cases reports in patients with dysferlinopathy (Bruss et al., 2004; Li et al., 2014; Zhu et al., 2015). In one case described in the literature, the elevation of transaminases was noted 3 years before the weakness appeared (Li et al., 2014) in the present study there was a weak negative correlation between CK, AST and ALT levels, respectively, with duration of disease.

This finding, along with elevated CK, can be used as a screening method to identify non-symptomatic family members of those with confirmed dysferlin mutations (Cupler et al., 1998). The data in the present study suggest that the persistent mild increase in liver enzymes is a phenomenon explicitly related to dysferlinopathy more than other subtypes of LGMD. But unfortunately, the evidence in the literature to support such a theory is lacking. CK levels are highly elevated in many autosomal recessive LGMD (Mahmood and Jiang, 2014). Notably high CK levels in dysferlinopathy are usually correlated with disease activity (Mahmood and Jiang, 2014). In the present study, all subjects had very high CK levels on more than one occasion, ranging from 302 IU/L to 21,483 IU/L (Table 1).

The cardiovascular system is rarely affected in dysferlinopathy. None of our patients showed prolonged QRS time. Only a few cardiac abnormalities were noted in our study, including congenital cardiac malformation (Ebstein anomaly) with right bundle branch block in one patient (F09B), ischemic cardiomyopathy with EF 20–30% in one (F10A), and prolonged QT interval in another subject (F12A). In the study by Nishikawa et al. (2016), out of 46 subjects who underwent ECG evaluation, a long QRS complex was noted in 40% with no significant association with age, disease duration, or respiratory function. An echocardiogram revealed no left ventricular dysfunction in all subjects in their study (Nishikawa et al., 2016).

Electromyographic changes in dysferlinopathy are particularly unique as many patients have various degrees of inflammatory changes during their disease. Additionally, neurogenic changes have been reported in dysferlinopathy. Same observation was noted in our study as myopathic changes with spontaneous activity were observed in the majority of the patients who underwent electrophysiological testing.

About 38.2% of the subjects in the present study had pelvic MRI. Literature review on the radiological features of patients with mutations in the DYSF gene has mainly focused on studying the lower limbs (Díaz-Manera et al., 2015). The two most common phenotypes of dysferlinopathy, Limb-girdle muscle dystrophy and Miyoshi Myopathy, are not differentiable using MRI (Kesper et al., 2009; Paradas et al., 2010; Díaz-Manera et al., 2015). According to the literature, gluteus minimus is most commonly involved muscle in dysferlin patients (Kesper et al., 2009; Díaz-Manera et al., 2015). However, adductor Magnus, semimembranosus, semitendinosus and gastrocnemius medial muscles are involved at the beginning of the disease (Diaz-Manera et al., 2018), with the first changes noted on imaging being a hyperintense signal in STIR sequence (Paradas et al., 2010; Jethwa et al., 2013; Díaz-Manera et al., 2015). These MRI findings are surprisingly present regardless of the phenotype (Paradas et al., 2010). The gracious and sartorius muscles were relatively spared until the end stages of the disease, as in many other muscular dystrophies (Paradas et al., 2010). Only two patients (F15A, F23C) in the present study showed the sartorius sparing phenomenon. Figure 7 t is also known that the rectus femoris muscle is spared until the very late stages of the disease forming, which is called (diamond sign) (Kesper et al., 2009; Paradas et al., 2010; Díaz-Manera et al., 2015). One subject (F23C) in the present study had shown sparing of the rectus femoris and hamstring muscle on imaging. In the legs, atrophy usually involves the gastrocnemius medialis and soleus muscle first and then progresses to affect the gastrocnemius lateralis and soleus (Díaz-Manera et al., 2015; Diaz-Manera et al., 2018) which was seen in almost all of the patient in the current study.

Limb-girdle muscular dystrophies mimicking polymyositis has been described frequently in the literature (Selva-O’Callaghan et al., 2006; Pimentel et al., 2008; Vinit et al., 2010; Angelini and Nigro, 2011; Jethwa et al., 2013). In the present study, muscle biopsy of nine subjects showed inflammatory changes mimicking the pathology seen in primary inflammatory myositis. These patients were erroneously diagnosed with Polymyositis and treated with a high-dose steroid. A double-blind placebo-controlled clinical trial of patients with dysferlinopathy treated with Deflazacort showed worsening of their muscle strength, advocating the current recommendation that steroids should not be administered in patients with dysferlinopathy (Walter et al., 2013). Similarly, in the present study, in dysferlinopathy subjects treated with a high dose steroid, there was a trend of muscle strength worsening and disease progression. This was particularly noted in one of the subjects (F21A) who lost ambulation and became wheelchair dependent within a few weeks after initiation of the steroid treatment. Sarcolemmal complement attack complex deposits on non-necrotic muscle fibers can help in differentiating genetically inherited muscular dystrophy with secondary inflammatory changes from primary inflammatory myositis (dermatomyositis, Polymyositis) (Spuler and Engel, 1998). This can be used as a hint to differentiate dysferlinopathy from Polymyositis.

Genetic analyses are assumed to diagnose around 99% of cases with known gene loci; however, sometimes, the gene locus is unknown (Mahmood and Jiang, 2014). The dysferlin gene encompasses 58 exons and also very large introns (KM, 1995; Aoki et al., 2001). Different mutations in the dysferlin gene have varying effects on the expression of the protein (Tagawa et al., 2003; Takahashi et al., 2003; Guglieri et al., 2008; Urtizberea et al., 2008). There has been an expression of the same homozygous mutation with variable phenotype presentation. It points out that other than genetic factors, environmental factors may also modify the phenotype (KM, 1995). Genotype is not a predictor of disease severity (Vilchez et al., 2005; Nguyen et al., 2007; van der Kooi et al., 2007; Paradas et al., 2010).

The DYSF gene is located at chr 2p13.2 with 58 exons (HGNC, 2021). It is involved in membrane regeneration and repair. It is predicted to encode a protein of 2,080 amino acids in an open reading frame (Aoki and Takahashi, 2004). It has been reported that more than 140 mutations in the *DYSF* gene causing 2B and more than 100 mutations causing Miyoshi myopathy have been identified [DYSF gene (dysferlin), 2020].

Novel *DYSF* gene mutations have also been reported with a possible founder effect (Cagliani et al., 2003). Cagliani et al. (2003), in their study, discussed the existence of a founder effect for the Arg959Trp mutation (2785C > T pathogenic variant) in the Italian population. In Spain population, Vilchez et al. (2005) in their study, observed the new R1905X *DYSF* founder mutation (C > T transition at 6,086 nucleotide position changing arginine into a stop codon). It resulted in three possible dysferlinopathy phenotypes without intrafamilial heterogeneity. In the present study, the mutation (c.164_165insA) was seen in 19 families indicating founder effect in Saudi population. This mutation has been reported before in other patients with Dysferlinopathy especially Miyoshi phenotype. Liu et al. (1998) also, in the current study, we couldn’t find any significant correlation between phenotype and genotype. In addition, the Dysferlin (*DYSF*) gene mutations have been distributed along the entire length of the gene without any hot spots.

Compound heterozygous DYSF gene mutations leading to dysferlinopathy had been reported in the literature (Nguyen et al., 2007; Li et al., 2017; Liu et al., 2018). Compound heterozygosity refers to the presence of two or more heterogeneous recessive alleles at a particular locus capable of causing genetic disease in a heterozygous state. Diers et al. (2007) reported a novel compound heterozygous mutation in 2007 in an adolescent LGMDR2 female with painful enlargement of calf muscles. In the current study, two patients were noted to have a heterozygous mutation (F16 A, F24A) one with MM phenotype and the other with LGMDR2 phenotype. Totally, Three novel variants were discovered namely c.5201A > T, c.4210_4211nsC and, c.1433del in the present study. In dysferlinopathy, there is a considerable variation in clinical presentation, phenotype characterizations, progression of disease, muscle involvement and genetic diagnosis. DYSF gene Mutations are linked with various clinical diagnosis like LGMDR2 and MMD. Initial clinical diagnosis cannot accurately predict the pattern of disease progression and rate of deterioration. There is a need for genetic testing and initial screening in new families for founder mutation.

The limitations of the present study include the cross-sectional nature of the study. Although it is a record-based study, the validity of the results depends on the accuracy of the patient details maintained in the records. The sampling frame of the study was the national cohort database for LGMD. Hence the generalizability of the present study results to the community is questionable because of the variability in clinical presentation due to the varied clinical phenotypes. This study for the first time will expand the land scope of our knowledge of Dysferlinopathy in Arab populations.

## Data Availability Statement

The datasets presented in this study can be found in online repositories. The names of the repository/repositories and accession number(s) can be found in the article/Supplementary Material.

## Ethics Statement

The studies involving human participants were reviewed and approved by Research Advisory Council (RAC). The patients/participants provided their written informed consent to participate in this study. Written informed consent was obtained from the individual(s) for the publication of any potentially identifiable images or data included in this article.

## Author Contributions

NA, RM, EC, HA-H, BM, AA, and SB made substantial contributions to the conception or design of the work and analysis and interpretation of data for the work, acquisition analysis and interpretation of data for the work, and revising articles critically for important intellectual content. NA, RM, EC, HA-H, IA, AA, MA, and DM contributed to the design of the work and analysis of data for the work. NA and SB drafted the work for important intellectual content and provided final approval of the version to be published. All authors have made a significant contribution to this article, have seen and approved the final article, and have agreed to its submission to the “Obesity Facts.”

## Conflict of Interest

The authors declare that the research was conducted in the absence of any commercial or financial relationships that could be construed as a potential conflict of interest.

## Publisher’s Note

All claims expressed in this article are solely those of the authors and do not necessarily represent those of their affiliated organizations, or those of the publisher, the editors and the reviewers. Any product that may be evaluated in this article, or claim that may be made by its manufacturer, is not guaranteed or endorsed by the publisher.

## Footnotes

1
http://www.affymetrix.com/support/technical/manuals.affx

2
http://dna-leeds.co.uk/autosnpa/

3
http://genome.ucsc.edu/
