# Supplementary material for: Clinical, Neurophysiological, Radiological, Pathological, and Genetic Features of Dysferlinopathy in Saudi Arabia
Source: Front Neurosci. 2022 Feb 22;16:815556. doi: 10.3389/fnins.2022.815556 (PMC8902167; doi:10.3389/fnins.2022.815556)
Supplement: Supplementary file 1 [file Table_1.pdf]

Supplementary Table: Sociodemographic, Clinical, Neurophysiological, Radiological, Pathological and Biochemical characteristics of Dysferlinopathies in Saudi Arabia (n=53)

S. No.	Family number	Gender /Age	Province/ Bedoin	Family History	Age of Onset	Initial Symptoms	Phenotype	Follow Up in years	Functional status	CK level (N <195 U/L)	AST (N <45)	ALT (N<45)	COMMENTS
**1**	F1 A	F/46	W/ YES	YES	14	Difficulty in getting from a chair	LGMD	6Y	Wheelchaired Since 30 years of age	1897	65	50	Asymmetric
**2**	F2 A	M/41	E/NO	NO	15	Difficulty standing from a chair, walking difficulty, and frequent falls	LGMD	6Y	Wheelchaired since 25years of age	9064	Not done	188	Received steroid for 7 years.
**3**	F3 A	F/48	C/YES	Yes	22	Exercise-related pain and mild proximal weakness	Proximodistal	10Y	Wheelchaired at age 29Y	1868	Not done	Not done	Marked intrafamilial variability
**4**	F3 B	M/40	C/YES	YES	17	Exercise-related pain and difficulties in running	MM	10Y	Wheelchaired at age 32	8713	Not done	Not done	Asymmetric. Marked intrafamilial variability
**5**	F 3 C	M/35	C/YES	YES	16	Proximal muscle weakness and backache	Proximodistal	10Y	Wheelchaired at age 25	9500	Not done	Not done	Marked intrafamilial variability
**6**	F3 D	F/34	C/YES	YES	17	Difficulties in getting from a chair	LGMD	10Y	Ambulating	10000	Not done	Not done	Asymmetric. Marked intrafamilial variability
**7**	F4 A	M/26	E/NO	NO	12	Difficulty walking up the stairs	LGMD	5Y	Ambulating	5000	78	78	Wrongly diagnosed as Becker for many years.

													Mild phenotype & Slowly progressive
**8**	F 5A	M/41	SW/NO	YES	19	Distal weakness	MM	8Y	Ambulating with aid	8459	95	96	Asymmetric. Marked intrafamilial variability
**9**	F5 B	M/33	SW/NO	YES	17	Gait problems and difficulties in getting from a chair	LGMD	8Y	Ambulating with aid	8506	Not don e	Not done	Marked intrafamilial variability
**10**	F6 A	F/42	W/NO	YES	15	Lumbar back pain and Progressive Lower limb weakness	MM	5 Y	Ambulating	7387	76	81	
**11**	F7 A	M/29	C/YES	YES	17	Distal weakness and difficulties in getting from a chair	MM	6Y	Ambulating with aid	1271 5	134	164	Asymmetric. Received steroid for 4 years
**12**	F7 B	F/27	C/YES	YES	16	Weakness on walking on toes	MM	6Y	Ambulating with aid	6000	Not don e	Not done	Diabetes
**13**	F 8 A	M/38	SW/NO	NO	17	Difficulty playing football & frequent injuries during the game	MM	13Y	Ambulating with aid	9120	Not don e	238	
**14**	F9 A	F/39	W/YES	YES	16	Exercise-related pain. Joint pain and weakness with fine motor hand movement	MM	5Y	Ambulating	1336	62	68	Wrongly diagnosed as psoriatic arthritis, then diagnosis changed to dermatomyositi s. Received

						and knee-buckling.							adalimumab, prednisone, methotrexate, and IVIG for 4 years with no improvement.
**15**	F9 B	F/34	W/YES	YES	25	Gradually progressive difficulty climbing stairs	LGMD	4	Ambulating	2099	70	81	S/P Ebstein anomaly repair.
**16**	F10 A	M/54	W/NO	YES	17	Back pain. Shaking arms when carrying heavy objects.	MM	6 Y	Ambulating.	2341	70	90	A mild form of Muscular Dystrophy. intrafamilial variability
**17**	F10 B	F/51	W/NO	YES	18	Lower limb weakness	Proximodist al	4 Y	Wheel-chaired since 31 years of age	3500	no	no	intrafamilial variability
**18**	F11 A	M/27	W/NO	No	16	Asymptomati c. Hyper CK	NO Symptom	4	Ambulating.	4284	102	186	N/A
**19**	F12A	F/39	W/YES	NO	10	Difficulty climbing stairs	LGMD	8	Ambulatory with walking aid	5557	84	127	Hypothyroid
**20**	F13 A	M/32	W/YES	YES	22	Difficulty carrying heavy objects	MM	4Y	Ambulatory with walking aid	1000 0	113	158	
**21**	F 13 B	F/37	W/YES	YES, but not geneticall y tested	20	Difficulty climbing stairs	MM	4	Wheel chaired at the age of 31	1100 0	Not don e	Not done	
**22**	F14 A	M/44	C/YES	YES	18	Back pain and lower extremity weakness	MM	28	Wheel-chaired since 33 years of age	8000	62	102	

**23**	F14 B	M/36	C/YES	YES	18	Back pain and lower extremity weakness	MM	18	Wheel chaired At the age of 30	1000 0	60	100	
**24**	F14 C	M/34	C/YES	YES	16		MM	16	Ambulatory with walking aid	1000 0	65	109	
**25**	F15 A	F/36	W/NO	NO	14	Weakness on walking	MM	11 Y	Ambulatory with walking aid	7700	103	172	
**26**	F16 A	F/56	SW/NO	YES	20	Distal weakness	MM	26	Wheel-chaired since 37 years of age	3750	84	64	Pancreatitis and Pulmonary Embolism
**27**	F 16 B	F/50	SW/NO	YES	17	Distal weakness and difficulties in getting from a chair	MM	16 Y	Wheelchaire d at the age of 35	7841	53	119	
**28**	F17 A	M/37	W/YES	NO	19	Difficulty getting up from a chair	MM	9Y	Ambulatory with walking air	9959	75	99	
**29**	F18 A	F/34	SW/NO	YES	17	Noticed she has difficulty standing on her toes and walking. Progressed to difficulty standing up from chair and difficulty swallowing??	Proximodist al	8 Y	Ambulatory with walking air	4567	98	81	
**30**	F18 B	M/36	SW/NO	YES	13	Difficulty climbing stairs and walking with back pain	MM	8Y	Wheelchair since 26 years of age due to frequent falls and serious	8000	91.2	159.6	Deltoid muscle hypertrophy. intrafamilial variability

									ligament injuries of both knees.				
**31**	F18 C	F/35	SW/NO	YES	14	Difficulty running because of knees instability and exercise intolerance	Proximodist al	8Y	Wheel-chair since 27 years of age	3600	135	137	Deltoid hypertrophy. intrafamilial variability ESR 26H Deltoid hypertrophy
**32**	F19 A	F/39	W/YRD	NO	15	Difficulty walking & climbing and descending stairs. Difficulty weakness in upper extremity	MM	9Y	Ambulating	1929	27	38	
**33**	F 20 A	M/39	W/NO	NO	25	Difficulty standing. Diagnosed initially with Polymyositis and received IVIG for 2 years.	LGMD	8Y	Ambulating with aids	4504	67	100	
**34**	F21 A	M/30	C/YES	NO	17	Incidental high CK and mildly elevated liver enzymes for investigation for acne. Diagnosed wrongly as Polymyositis. Given high dose steroid	N/A	6Y	Wheel-chaired since 19 years of age.	2148 3	318	656	Diagnosed wrongly as Polymyositis. Given high dose steroid then tapering dose. Deltoid sparing

						then tapering dose. Weakness 1 week after pulse therapy.							
**35**	F22 A	F/38	E/YES	YES	25	Difficulty walking stairs and difficulty with walking on wearing heels and frequent falls	Proximodist al	1YR	Ambulating	5000			Deltoid sparing
**36**	F22 B	M/55	E /YES	YES	24	Distal more than proximal weakness	MM	1YR	Wheel-chaired since around 30 years of age	1900	Not don e	Not done	
**37**	F22 C	M/49	E/YES	YES	21	Difficulty climb the stair Difficulty in standing for a long period	LGMD	1YR	Wheel-chaired since around 30 years of age	2890	Not don e	Not done	
**38**	F22 D	F/45	E/YES	YES	20	Difficulty climb the stair Difficulty in standing for a long period	LGMD	1YR	Wheel-chaired since around 30 years of age	1256	Not don e	Not done	
**39**	F22 E	M/50	E/YES	YES	23	Difficulty climb the stair Difficulty in standing for a long period	LGMD	1YR	Wheel-chaired since around 30 years of age	3000	Not don e	Not done	
**40**	F23 A	F/61	C/YES	YES	17	Difficulty climbing stairs	MM	9Y	Wheelchaire d since 30 years of age	3000	Not don e	Not done	

**41**	F23B	F/54	C/YES	YES	18	Difficulty climbing stairs	MM	9Y	Wheelchaired since 32 years of age	1500	Not done	Not done	
**42**	F23 C	F/53	C/YES	YES	13	Lower back and knee pain. Knee buckling. Frequent falls.	Proximodistal	9Y	Wheelchaired since 35 years of age	1916	56	53	Wrongly diagnosed Polymyositis and received steroid, methotrexate, IVIG, and Rituximab for 8 years. Deltoid sparing. Hyper-extension of the knees
**43**	F24 A	F/38	E/NO	NO	20	Limping and recurrent fall	LGMD	8Y	Ambulating with aid	8496	45	133	Hypertrophied deltoid muscle
**44**	F25 A	M/34	C/No	No	22	Difficulty climb the stair Difficulty in standing for a long period	Proximodistal	9Y	Ambulating	7858	114	171	
**45**	F26 A	F/31	S/NO	NO	15	Difficulty walking stairs	LGMD	12	Ambulating	302	66	63	Dermatomyositis
**46**	F27 A	M/22	S/NO	NO	14	Difficulty walking stairs	MM	2Y	Ambulating	10387	215	305	Heterozygous VUS. gene
**47**	F28 A	M/33	C/YES	YES	15	Difficulty in running	MM	12	Ambulating	10372	194	126	
**48**	F29 A	M/41	W/YES	YES	18	Recurrent falls	MM	19	Ambulating	12529	83	198	
**49**	F30A	M/41	C/yes	NO	20	Difficulty walking stairs	LGMD	16	Ambulating	1529	30	57	
**50**	F31 A	F/54	C/NO	NO	23	Difficulty walking stairs	LGMD	16	Ambulating	1005	33	24	
**51**	F32 A	M/37	C/YES	YES	14	Unable to run	LGMD	13	Ambulating	10000	Not done	186	

**52**	F33 A	M/32	C/YES	YES	22	Unable to run	MM	1	Ambulating	1114 1	128	145	More weakness in the upper limb
**53**	F33B	F/29	C/YES	YES	18	Unable to run	MM	1	Ambulating	8856	60	91	
